# Resveratrol protects against early polymicrobial sepsis-induced acute kidney injury through inhibiting endoplasmic reticulum stress-activated NF-κB pathway

**DOI:** 10.18632/oncotarget.16860

**Published:** 2017-04-05

**Authors:** Nian Wang, Li Mao, Liu Yang, Jiang Zou, Ke Liu, Meidong Liu, Huali Zhang, Xianzhong Xiao, Kangkai Wang

**Affiliations:** ^1^ Department of Pathophysiology, Xiangya School of Medicine, Translational Medicine Center of Sepsis, Central South University, Changsha, 410078, Hunan, China

**Keywords:** sepsis, acute kidney injury, endoplasmic reticulum stress, inositol-requiring enzyme 1, nuclear factor-κB

## Abstract

Resveratrol, a polyphenol compound derived from various edible plants, protects against sepsis-induced acute kidney injury (AKI) via its anti-inflammatory activity, but the underlying mechanisms remain largely unknown. In this study, a rat model of sepsis was established by cecal ligation and puncture (CLP), 30 mg/kg resveratrol was intraperitoneally administrated immediately after the CLP operation. HK-2 cells treated by 1 μg/ml lipopolysaccharide, 0.2 μM tunicamycin, 2.5 mM irestatin 9389 and 20 μM resveratrol were used for *in vitro* study. The results demonstrated that resveratrol significantly improved the renal function and tubular epithelial cell injury and enhanced the survival rate of CLP-induced rat model of sepsis, which was accompanied by a substantial decrease of the serum content and renal mRNA expressions of TNF-α, IL-1β and IL-6. In addition, resveratrol obviously relieved the endoplasmic reticulum stress, inhibited the phosphorylation of inositol-requiring enzyme 1(IRE1) and nuclear factor-κB (NF-κB) in the kidney. *In vitro* studies showed that resveratrol enhanced the cell viability, reduced the phosphorylation of NF-κB and production of inflammatory factors in lipopolysaccharide and tunicamycin-induced HK-2 cells through inhibiting IRE1 activation. Taken together, administration of resveratrol as soon as possible after the onset of sepsis could protect against septic AKI mainly through inhibiting IRE1-NF-κB pathway-triggered inflammatory response in the kidney. Resveratrol might be a readily translatable option to improve the prognosis of sepsis.

## INTRODUCTION

Sepsis, an infection induced systemic inflammatory response syndrome (SIRS), has become the leading cause of mortality in the intensive care unit (ICU) worldwide [1, 2]. It is caused by various etiological factors and can result in multiple organ dysfunctions (MODS) because of lasting and uncontrolled inflammatory response. So far, the clinical management of sepsis is still limited to symptomatic and supportive treatments that aim to maintain the homeostasis of important organs and prevent the further infection. In addition, targeted therapies on some specific inflammatory factors show little improvement on the prognosis of sepsis, the mortality still remains unacceptably high. Kidney is one of the organs most easily damaged by sepsis. Acute kidney injury (AKI) is a common and potentially fatal complication of sepsis and develops in about 50% of patients with severe sepsis. Once the patients suffer from severe sepsis complicated with AKI, the mortality can rise up to 70% [3]. Prompt and effective amelioration of AKI can contribute to improve the prognosis of sepsis. Therefore, exploring novel targets and strategies that can ameliorate septic AKI is still of great importance.

To date, the underlying mechanisms implicated in sepsis-induced AKI haven't been fully elucidated. It has been proved that excessive inflammatory response, intrarenal hemodynamic changes, blood coagulation dysfunction, microvascular endothelial dysfunction and renal tubular epithelial cell injury are all implicated in this process [4]. Sepsis-induced overwhelming inflammatory response results in the production and release of a large amount of cytokines such as tumor necrosis factor-α (TNF-α), interleukin-1β (IL-1β), interleukin-6 (IL-6), inflammatory mediators and chemokines, which is considered as the direct trigger of septic AKI and eventually lead to renal tubular epithelial cell injury [5].

Nuclear factor-κB (NF-κB) is a key transcriptional regulator of inflammatory genes, including TNF-α, IL-1β and IL-6, etc. Accumulating evidences suggest that the activation of NF-κB can promote the transcription of these pro-inflammatory genes, trigger the inflammation cascade and play an important role in a variety of inflammatory diseases [6–9]. It was firstly reported by H L Pahl et al that the activation of NF-κB (p65) was mediated by the signaling pathway from endoplasmic reticulum (ER) [10]. ER, an important organelle responsible for the protein synthesis, folding and maturation, is extremely sensitive to different kinds of endogenous and exogenous harmful stimuli, such as infection, burn, trauma and so on, which can further result in the disturbance of ER lumen called ER stress. It has been proved that the activation of three ER resident transmembrane proteins, PKR-like ER stress kinase (PERK), activating transcription factor 6(ATF6) and inositol-requiring enzyme 1(IRE1) mediate the ER stress signaling pathway and are implicated in the development of various diseases, including sepsis [11–13]. Moreover, the activation of NF-κB(p65) was regulated by these three branch pathways mentioned above. Therefore, amelioration of ER stress may be beneficial to inhibit the early inflammatory response and further improve the prognosis of septic AKI.

Resveratrol(trans-3,5,4′-trihydroxystilbene) is a polyphenol compound derived from various edible plants such as grape, peanut, berry, veratrum and so on. It has multiple biological activities, including anti-inflammation, antioxidation, antitumor, insulin sensitization, etc. It has been proved to have beneficial effect on a variety of diseases including sepsis-induced AKI via its anti-oxidant and anti-inflammatory properties [14, 15], but the underlying mechanism remains obscure. It should be noted that resveratrol has been proved to be useful in treating various experimental disease models through relieving ER stress [16–18]. However, whether the inhibition of ER stress-induced NF-κB activation and inflammatory response are involved in the protective effect of resveratrol on sepsis-induced AKI is largely unknown. In this study, attention had been focused on a better understanding of the molecular mechanisms contributing to sepsis-induced AKI and the protective effect of resveratrol.

## RESULTS

### Resveratrol improved the survival, renal function and structure damage of rats with polymicrobial sepsis

**Figure 1:**
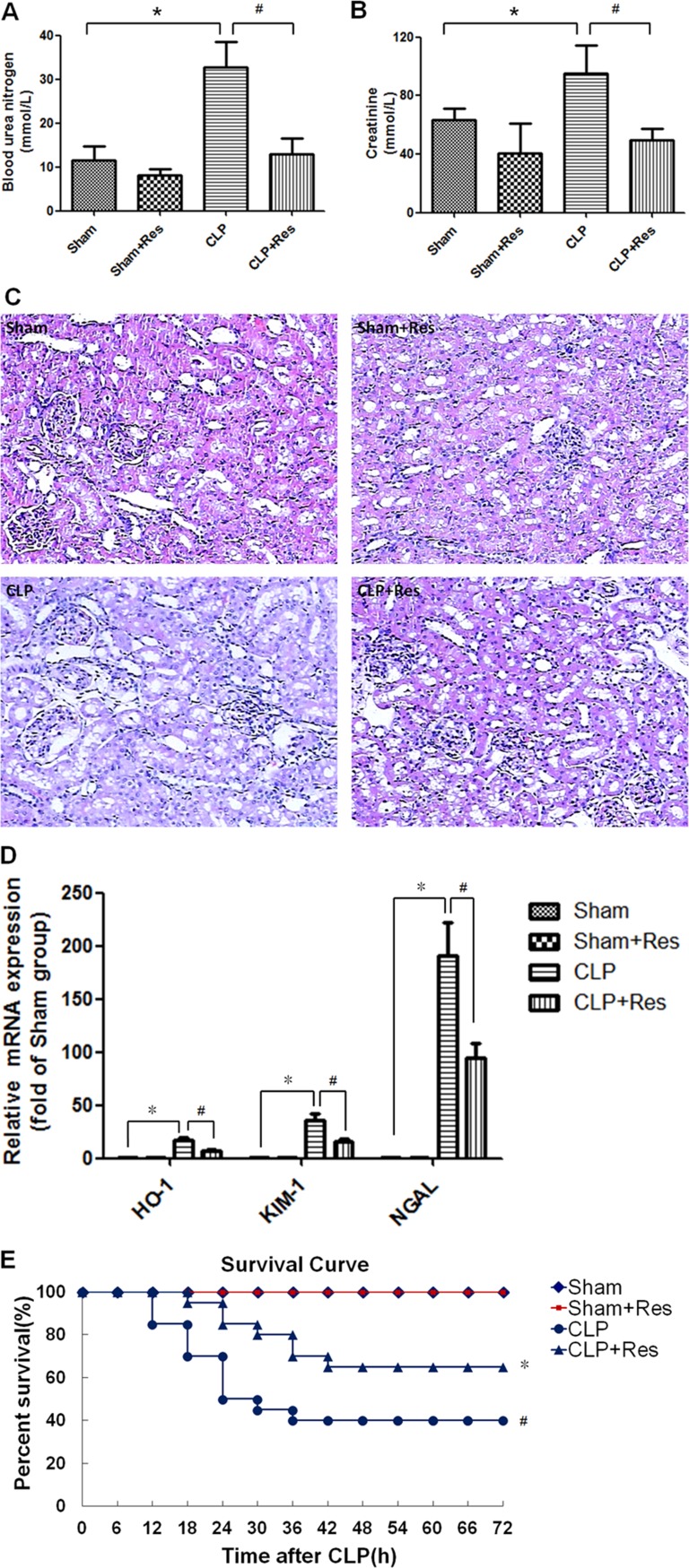
Resveratrol improved the survival, renal function and structure damage of rats with polymicrobial sepsis-induced AKI A rat model of polymicrobial sepsis-induced AKI was established by CLP operation, 30 mg/kg resveratrol was intraperitoneally administrated immediately after CLP operation. (**A** and **B**)At 12 hours after CLP operation, the blood urea nitrogen (A) and serum creatinine (B) levels were detected (*n* = 5 per group). (**C**) At 12 hours after CLP operation, the renal morphology was detected by HE staining (×100). (**D**) At 12 hours after CLP operation, the mRNA expressions of HO-1, KIM-1 and NGAL in the kidney were detected by RT-PCR (*n* = 5 per group). (D) Kaplan–Meier test was performed to analyze the survival of rats (*n* = 20 per group). Note: *p < 0.05 *vs*. Sham group, ^#^*p* < 0.05 *vs*. CLP group.

To determine the effect of resveratrol on sepsis-induced AKI, a rat model of polymicrobial sepsis was established by CLP operation. Compared with the sham-operated controls, the blood urea nitrogen and serum creatinine levels which were commonly used in clinic to reflect the renal function were increased 1~2 folds (32.82 ± 5.67 *vs*. 11.6 ± 3.15, 95.04 ± 19.09 *vs*. 63.3 ± 8.13) at 12 hours after CLP operation (*p* < 0.05) (Figure 1A, 1B). Meanwhile, the kidney tissue of CLP-operated rats showed obvious edema of renal proximal tubular epithelial cells, narrowing of renal proximal tubular lumina and derangement of glomerular vessels as well as sporadic inflammatory cells infiltration (Figure 1C). Heme oxygenase-1 (HO-1), kidney injury molecule-1(KIM-1) and neutrophil gelatinase-associated lipocalin(NGAL) were considered as the markers of renal tubular injury. In this study, compared with the sham-operated controls, the mRNA expressions of HO-1, KIM-1 and NGAL in the kidney of CLP-operated rats enhanced 16.63 ± 2.43, 36.4 ± 6.16, 190.33 ± 31.47 folds respectively at 12 hours after CLP operation (*p* < 0.05) (Figure 1D). Moreover, the 72 h-survival rate of CLP-operated rats was decreased to 40% (Figure 1E). By contrast, resveratrol decreased the blood urea nitrogen and serum creatinine levels as well as the renal mRNA expressions of HO-1, KIM-1 and NGAL, enhanced the survival rate to 65% and restored the renal structure damage of CLP-operated rats. These results suggested that resveratrol could alleviate polymicrobial sepsis-induced early AKI and improve the survival of sepsis.

### Resveratrol decreased the renal production and release of pro-inflammatory cytokines in rats with polymicrobial sepsis

**Figure 2:**
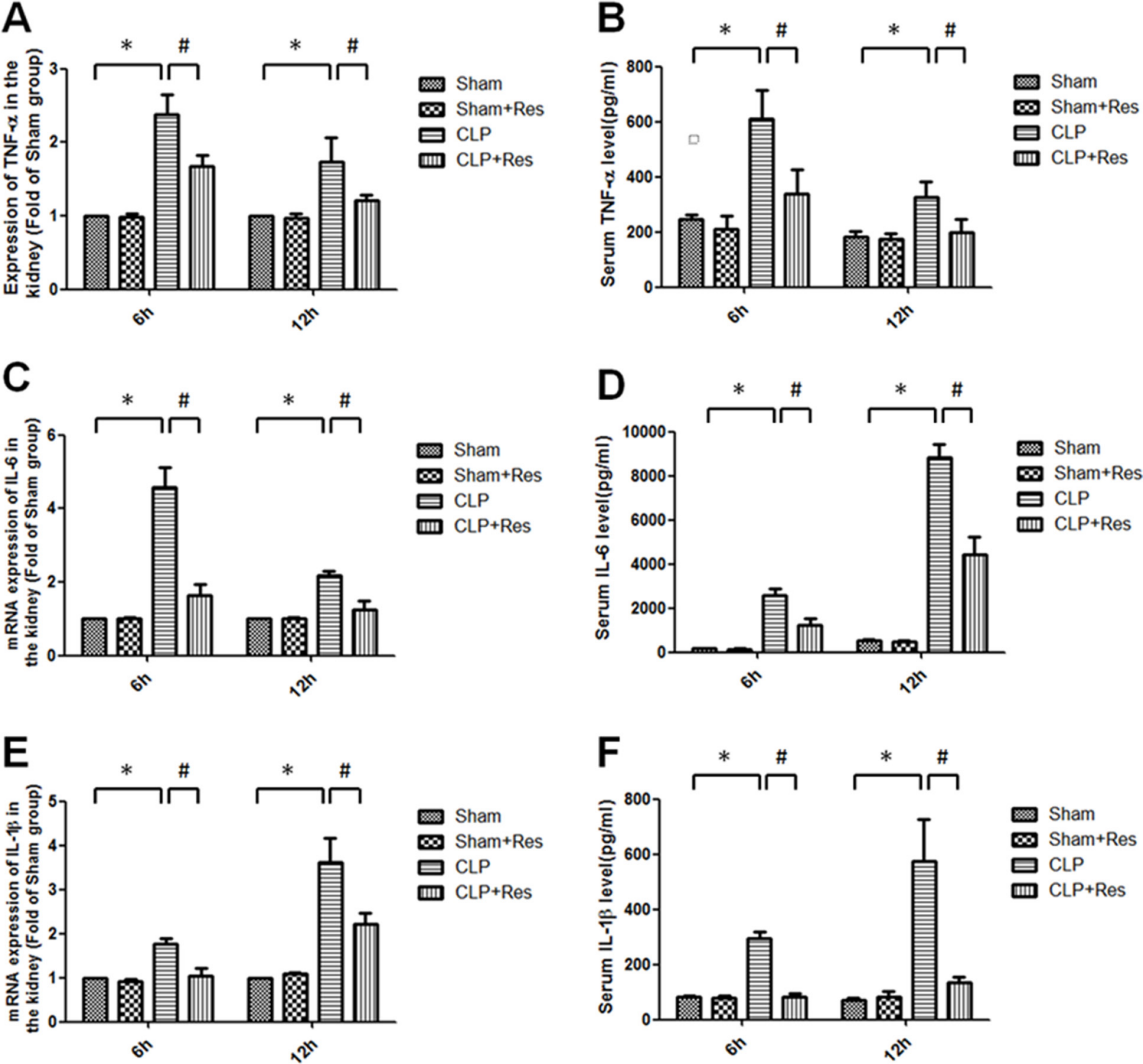
Resveratrol decreased the renal production and release of pro-inflammatory cytokines in rats with polymicrobial sepsis The renal mRNA expressions of TNF-α (**A**) IL-1β (**C**) IL-6 (**E**) and serum levels of TNF-α (**B**) IL-1β (**D**) IL-6 (**F**) at 6, 12 hours after CLP operation were measured by RT-PCR and ELISA respectively. Note: Data were shown as mean ± S.E.M (*n* = 5 per group, *p < 0.05 *vs*. Sham group, ^#^*p* < 0.05 *vs*. CLP group).

As local and systemic inflammatory response were considered as the direct and initial factors of septic AKI, the renal TNF-α, IL-1β, IL-6 mRNA expressions and serum TNF-α, IL-1β, IL-6 levels were measured. It was shown that the renal TNF-α, IL-1β, IL-6 mRNA expressions increased 2.38 ± 0.27, 1.77 ± 0.11, 4.58 ± 0.55 folds and 1.74 ± 0.33, 3.62 ± 0.55, 2.18 ± 0.13 folds respectively at 6, 12 hours after CLP compared with the sham-operated controls (Figure 2A, 2C, 2E), while the serum levels of TNF-α (609.87 ± 103.9 *vs*. 248.57 ± 12.55; 328.56 ± 53.9 *vs*. 183.08 ± 17.6 pg/ml), IL-1β (294.64 ± 25.87 *vs*. 82.91 ± 4.75; 576 ± 148.87 *vs*. 69.68 ± 10.28 pg/ml), IL-6 (2595.86 ± 295.64 *vs*. 162.45 ± 12.56; 8815.04 ± 603.166 *vs*. 541.61 ± 25.98 pg/ml) were all substantially increased at 6, 12 hours after CLP operation (Figure 2B, 2D, 2F). Interestingly, resveratrol significantly reduced about 40%–60% in the increase of these pro-inflammatory factors. Taken together, resveratrol inhibited the renal and systemic inflammatory response in rats with polymicrobial sepsis at the early stage.

### Resveratrol inhibited the expression and activation of NF-κB in the kidney of rats with polymicrobial sepsis

**Figure 3:**
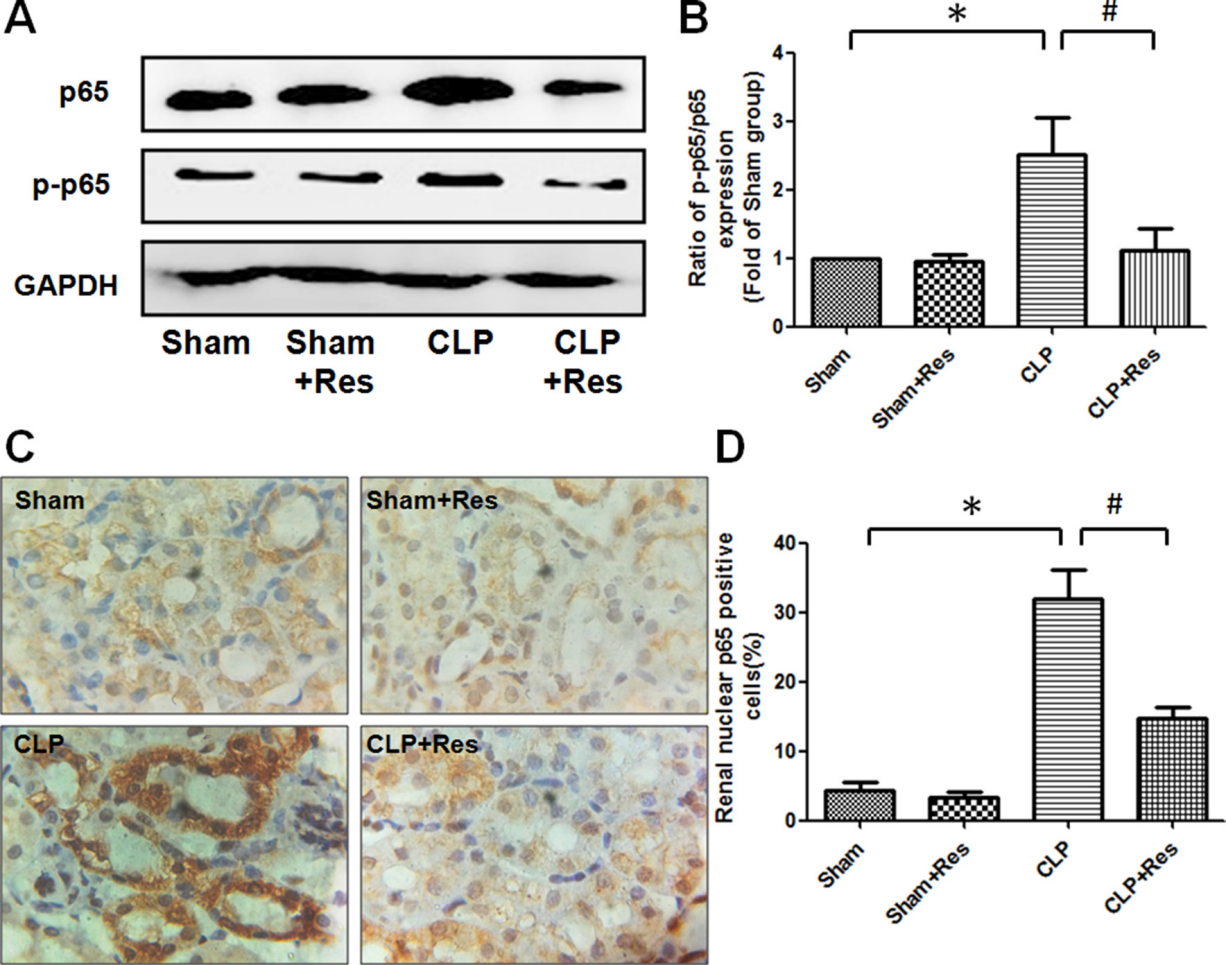
Resveratrol inhibited the expression and activation of NF-κB in the kidney of rats with polymicrobial sepsis (**A**) At 12 hours after CLP operation, the expressions of NF-κB (p65) and phosphorylated p65 in the kidney were detected by western blotting. (**B**) The ratio of phosphorylated p65/total p65 (p-p65/p65) was calculated. (**C**) The renal expression of p65 at 12 hours after CLP operation was detected by immunohistochemical staining (×200). (**D**) The percentage of renal nuclear p65-positive cells was calculated. Note: Data were shown as mean ± S.E.M (*n* = 5 per group, *p < 0.05 *vs*. Sham group, ^#^*p* < 0.05 *vs*. CLP group).

To investigate the mechanism by which resveratrol inhibited the inflammatory response in the kidney during sepsis, the expression and activation of NF-κB (p65) which was a key regulator of pro-inflammatory factors transcription were detected. It was shown that both the expressions of p65 and phosphorylated p65 (p-p65) were obviously increased in the kidney of rats at 12 hours after CLP operation as compared with the sham-operated controls, the ratio of p-p65/p65 increased 2.51 ± 0.55 folds, while resveratrol partially decreased the expressions of p65 and phosphorylated p65 and restored about 50% of the increase in the ratio of p-p65/p65 (Figure 3A, 3B). Immunohistochemical staining demonstrated that the percentage of renal nuclear p65-positive cells of rats at 12 hours after CLP operation was 32 ± 4.04% (Figure 3C, 3D), which was significantly higher than that of the sham-operated controls (4.33 ± 1.20%) and remarkably reduced by the treatment of resveratrol (14.67 ± 1.76%). These data indicated that NF-κB (p65) activation was implicated in the development of polymicrobial sepsis-induced AKI, while resveratrol might alleviate the renal inflammatory response during sepsis-induced AKI through the inhibition of NF-κB (p65) activation.

### Resveratrol relieved ER stress in the kidney of rats with polymicrobial sepsis

**Figure 4:**
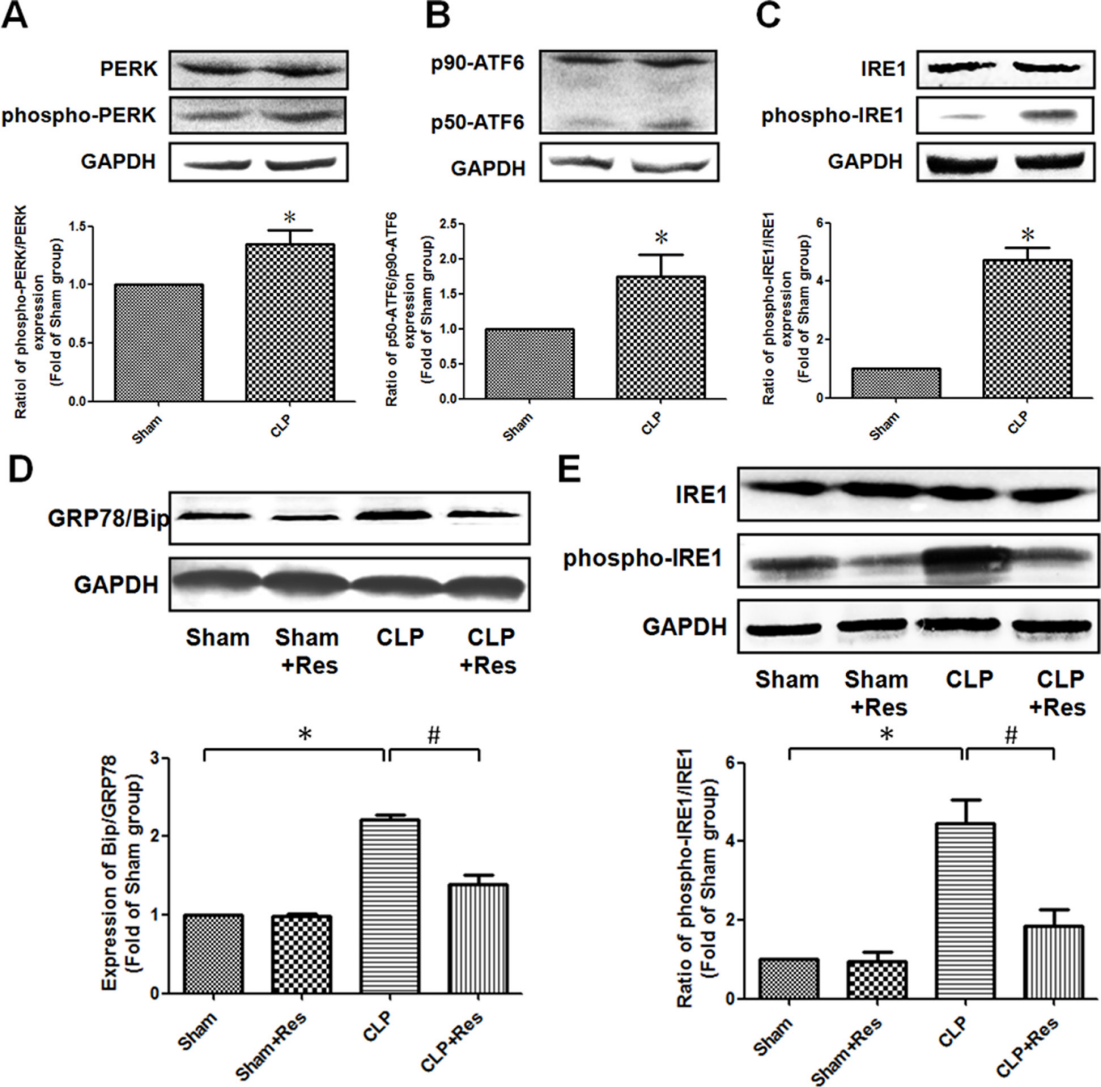
Resveratrol relieved ER stress in the kidney of rats with polymicrobial sepsis (**A**–**C**) At 12 hours after CLP operation, the expressions of PERK (A), ATF6 (B) and IRE1 (C) in the kidney were detected by western blotting. (**D** and **E**) At 12 hours after CLP operation, the expressions of GRP78/Bip, IRE1 and phosphorylated (ser724) IRE1 in the kidney were detected by western blotting. Note: Data were shown as mean ± S.E.M (*n* = 5 per group, *p < 0.05 *vs*. Sham group, ^#^*p* < 0.05 *vs*. CLP group).

To determine whether ER stress was implicated in the protective effect of resveratrol on septic AKI, the expression of glucose-regulated protein 78 (GRP78/Bip) that was considered as an important mediator and marker of ER stress was firstly detected. It was found that GRP78/Bip expression in the kidney of CLP-operated rats at 12 hours after CLP operation was increased 2.21 ± 0.07 folds as compared with the sham-operated controls (Figure 4D). In addition, the ratio of phosphorylated PERK, cleaved ATF6 (p50-ATF6) and phosphorylated IRE1 were elevated 1.35 ± 0.12, 1.75 ± 0.31 and 4.73 ± 0.41 folds respectively in comparison to the sham-operated controls (Figure 4A, 4B, 4C). These results suggested that ER stress was involved in the development of septic AKI. What was worthy to be mentioned was that the activation of IRE1 was more obviously than ATF6 and PERK in the kidney of CLP-operated rats. Therefore, the activation of ER stress (especially IRE1 pathway) might mainly contribute to the development of sepsis-induced AKI. It should be noted that resveratrol significantly attenuated the increase of GRP78/Bip and phosphorylated (ser724) IRE1 expressions (1.39 ± 0.12 *vs*. 2.21 ± 0.07; 1.85 ± 0.43 *vs*. 4.45 ± 0.62) in the kidney of CLP-operated rats (Figure 4E). Taken together, these data demonstrated that the inhibition of IRE1 pathway was mainly involved in the protective effect of resveratrol on septic AKI.

### Resveratrol alleviated the inflammatory response in lipopolysaccharide(LPS) and tunicamycin-induced HK-2 cells through the activation of IRE1

**Figure 5:**
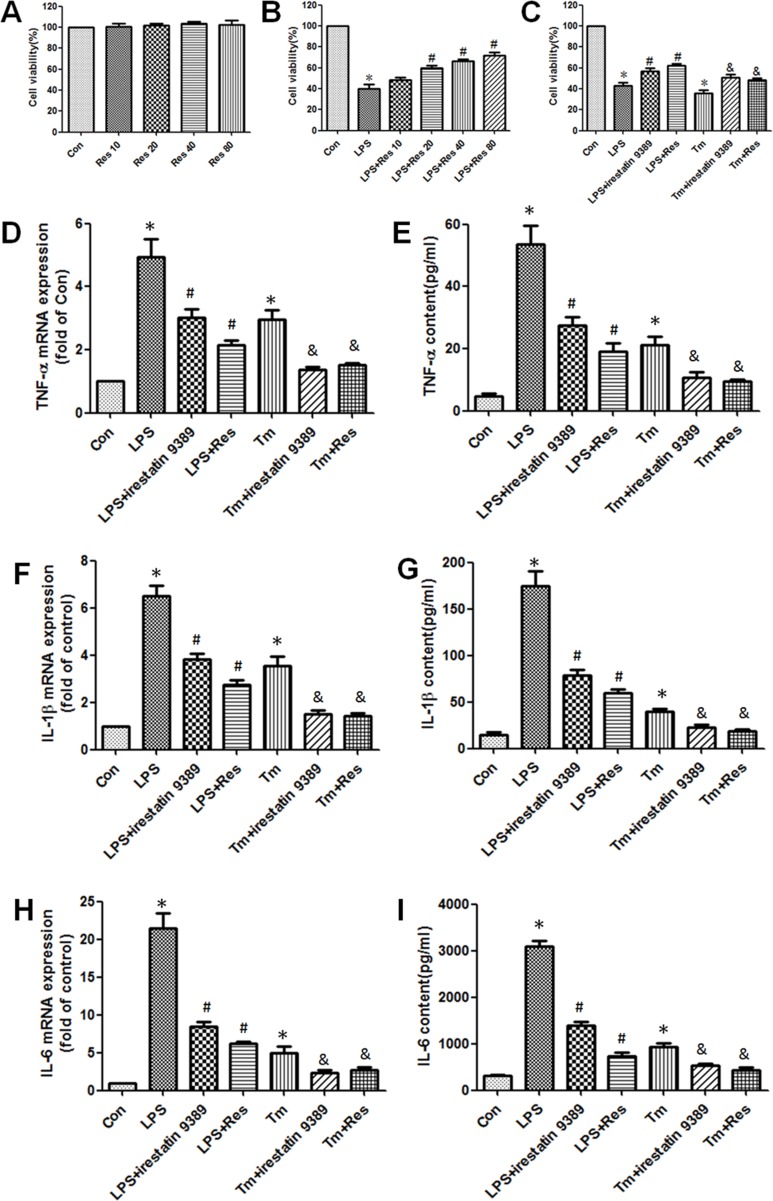
Resveratrol alleviated the inflammatory response in LPS and tunicamycin-induced HK-2 cells through inhibiting the activation of IRE1 (**A**) Effect of different concentrations (10, 20, 40, 80 μM) of resveratrol on the viability of HK-2 cells under normal condition was detected by MTT assay. Data were shown as the percentage of viable cells compared with the control group which was considered as 100%. (**B**) Effect of different concentrations (10, 20, 40, 80 μM) of resveratrol on the viability of LPS (1 μg/ml) -induced HK-2 cells was detected by MTT assay. Data were shown as mean ± S.E.M (*n* = 5 per group, *p < 0.05 *vs*. Con group, ^#^*p* < 0.05 *vs*. LPS group). (**C**) Effect of resveratrol on the viability of LPS and tunicamycin-induced HK-2 cells was detected by MTT assay. HK-2 cells were treated by LPS (1 μg/ml) or tunicamycin (0.2 μM) with or without irestatin 9389 (2.5 mM) and resveratrol (20 μM) for 12 hours. (**D**–**I**) Effect of resveratrol on the mRNA expressions of TNF-α (D), IL-1β (F), IL-6 (H) in HK-2 cells and the contents of TNF-α (E), IL-1β (G), IL-6 (I) in culture supernatant of LPS and tunicamycin-induced HK-2 cells were detected by RT-PCR and ELISA respectively. HK-2 cells were treated by LPS (1 μg/ml) or tunicamycin (0.2 μM) with or without irestatin 9389 (2.5 mM) and resveratrol (20 μM) for 12 hours. HK-2 cells treated by vehicle alone were used as the control group (Con). Data were shown as mean ± S.E.M (*n* = 5 per group, *p < 0.05 *vs*. Con group, ^#^*p* < 0.05 *vs*. LPS group, ^&^*p* < 0.05 vs. Tm group).

As IRE1 has been proved to be closely related to the regulation of inflammatory response during ER stress, IRE1 specific inhibitor irestatin 9389 and ER stress inducer tunicamycin were used to indentify whether IRE1 mediated the anti-inflammatory effect of resveratrol. Firstly, MTT assay was used to analyze the cytotoxicity of resveratrol. Different concentrations of resveratrol were treated on HK-2 cells under normal condition, it was shown that resveratrol had no significant effect on the viability of HK-2 cells (Figure 5A). It suggested that resveratrol had no cytotoxicity on the HK-2 cells in a certain range of concentrations. Furthermore, resveratrol (20~80 μM) enhanced the viability of HK-2 cells that was decreased by LPS in a concentration dependent manner (Figure 5B). Then 20 μM resverarol was used to treat HK-2 cells induced by LPS (1 μg/ml) and tunicamycin (0.2 μM). The results demonstrated that the decrease in viability of LPS and tunicamycin-induced HK-2 cells were obviously enhanced by resveratrol and irestatin 9389 (Figure 5C). Additionally, both LPS and tunicamycin substantially elevated the TNF-α, IL-1β, IL-6 mRNA expressions in HK-2 cells and contents in the culture supernatant, while resveratrol and irestatin 9389 obviously restored these changes (Figure 5D–5H). These findings indicated that resveratrol might alleviate the inflammatory response in LPS-induced HK-2 cells through inhibiting the activation of IRE1.

### Resveratrol reduced the activation of NF-κB(p65) through inhibiting ER stress-activated IRE1 in LPS-induced HK-2 cells

**Figure 6:**
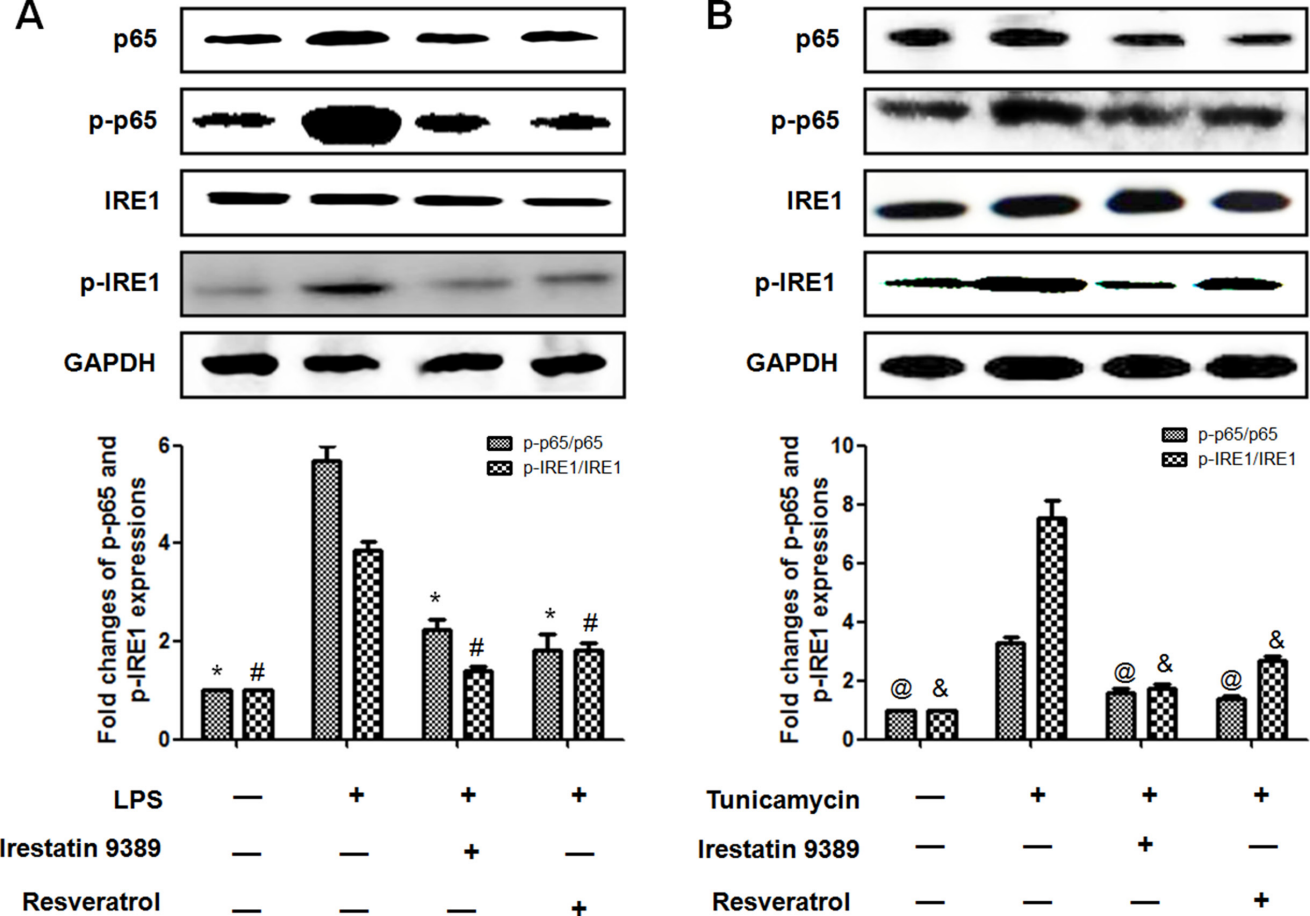
Resveratrol reduced the activation of NF-κB(p65) through inhibiting ER stress-activated IRE1 in LPS-induced HK-2 cells (**A**) Effect of resveratrol on LPS-induced activation of IRE1-p65 pathway in HK-2 cells. HK-2 cells were treated by LPS (1μg/ml) with or without irestatin 9389 (2.5 mM) and resveratrol (20 μM) for 12 hours. (**B**) Effect of resveratrol on the activation of IRE1-p65 pathway induced by tunicamycin (0.2 μM) in HK-2 cells. HK-2 cells were treated by LPS tunicamycin (0.2 μM)with or without irestatin 9389 (2.5 mM) and resveratrol (20 μM) for 12 hours. The expressions of p65, p-p65, IRE1 and p-IRE1 were detected by western blotting. Note: Data were shown as mean ± S.E.M (*n* = 5 per group, *p < 0.05, ^#^*p* < 0.05 *vs*. LPS group; ^@^*p* < 0.05, ^&^*p* < 0.05 *vs*. tunicamycin group).

To identify whether the protective effect of resveratrol was dependent on the inhibition of ER stress-activated IRE1-NF-κB (p65) pathway, both the expressions and activation of NF-κB (p65) and IRE1 were further determined. It was shown that LPS significantly enhanced the expressions of p65, p-p65 and p-IRE1, the effect was similar to the ER stress inducer tunicamycin. It should be noted that the ratio of p-p65/p65 and p-IRE1/IRE1 were both increased in LPS and tunicamycin-induced HK-2 cells, indicating that both p65 and IRE1 were activated. In contrast, resveratrol or irestatin 9389 obviously attenuated the expressions of p65, p-p65, p-IRE1 and the ratio of p-p65/p65 and p-IRE1/IRE1 in LPS and tunicamycin-induced HK-2 cells. These results indicated that resveratrol attenuated LPS–induced inflammatory response in the HK-2 cells through inhibiting the activation of IRE1-p65 pathway (Figure 6).

## DISCUSSION

Sepsis was newly defined as a life-threatening organ dysfunction (OD) caused by a dysregulated host response to infection in February, 2016 [19]. This new definition (sepsis 3.0) emphasized that OD was the crucial risk factor for the poor prognosis of sepsis, and timely treatment of OD should be prior to the control of infection. AKI was one kind of OD most commonly found in patients with severe sepsis and greatly contributed to the unacceptably high mortality of patients [3]. Hence, treatment of sepsis should be more focused on the septic AKI.

Inflammatory response was increasingly regarded as the central triggering factor to initiate sepsis-induced AKI [20]. Though resveratrol had been proved to be effective to alleviate septic AKI, the studies were mostly concentrated on the renal microcirculation and inflammatory response mediated by inflammatory cells at relatively late stage of sepsis (at least 18 hours after CLP or 20 hours after LPS administration), and resveratrol was administrated at least 2 hours after the induction of sepsis [14, 15, 21, 22]. However, once septic AKI occurred and developed, the disease condition would become more complex and more difficult to treat. Therefore, to elucidate the renoprotective effect of resveratrol on the early stage of sepsis and its underlying mechanisms were still of great importance and should be more focused on. In our study, early administration of resveratrol obviously improved the renal function, relieved the edema of renal tubular epithelial cells during sepsis. Though no obvious necrosis or apoptosis was found in the kidney of septic rats at 12 hours after CLP operation, the mRNA expressions of HO-1, KIM-1 and NGAL which were well-characterized markers of renal proximal tubule injury were obviously enhanced [23–25], while resveratrol substantially reversed these changes, suggesting that early administration of resveratrol was able to protect against sepsis-induced renal proximal tubule injury. Accordingly, resveratrol also significantly enhanced the survival rate of septic rats.

Generally speaking, innate inflammatory cells including macrophages, neutrophils and lymphocytes were considered as the first line of defense against different pathogens and main production resource of inflammatory cytokines. In recent years, several animal experiments showed that massive inflammatory cells infiltration and renal tubular epithelial cells apoptosis could be observed at 24 hours after CLP [26–28]. In our study, only sporadic inflammatory cells infiltration were found around glomerulus at 12 hours after CLP, suggesting that inflammatory response mediated by inflammatory cells might not play a leading role at the early stage of sepsis-induced AKI. It should be noted that other kinds of cells such as renal tubular epithelial cells were also found to have immunocompetence and can mediate immune and inflammatory responses [29–31]. Therefore, we thought that inflammatory response mediated by renal tubular epithelial cells might be earlier and more important than inflammatory cells in sepsis-induced AKI. Indeed, TNF-α, IL-1β, IL-6 mRNA expressions in the kidney were found to be greatly enhanced at 6 hours after CLP which was regarded as the early stage of sepsis [32, 33]. We speculated that inflammatory response occurred in the tubular epithelial cells at the early stage of sepsis contributed greatly to the occurrence of AKI. Moreover, the serum TNF-α, IL-1β, IL-6 levels were significantly increased in septic rats, while resveratrol decreased both the renal mRNA expressions and serum levels of TNF-α, IL-1β, IL-6. These findings suggested that early administration of resveratrol was beneficial to inhibit the systemic and local inflammation at the early stage of sepsis and subsequently prevent the dysfunction and injury of multiple organs. Then, how resveratrol attenuated the inflammatory response in the kidney during sepsis?

It had been reported in numerous studies that the biological properties of resveratrol were closely related to the inhibition of TLR4/NF-κB signaling pathway [34–36]. The activation of NF-κB (p65) was proved to play important roles in the development and progression of sepsis through enhancing the transcription of various pro-inflammatory cytokines including TNF-α, IL-1β, IL-6 [37]. But whether the protective effect of resveratrol on sepsis-induced AKI was also related to the inhibition of NF-κB (p65) activation was still unknown until now. In our study, the results demonstrated that the expression, phosphorylation and nuclear translocation of NF-κB (p65) which indicated the activation of NF-κB (p65) in the kidney of septic rats and LPS-induced HK-2 cells were robustly increased, while resveratrol obviously reversed these changes, suggesting that resveratrol might decrease the production of pro-inflammatory factors through inhibiting the activation of NF-κB in the kidney of septic rats.

NF-κB (p65) was mainly located in the cytoplasm and inactivated through binding with IκB under normal condition. The activation of NF-κB(p65) was mediated by the activation of IκB kinase (IKK) which could further phosphorylate IκB and induce the nuclear translocation of NF-κB (p65). In the cell nucleus, NF-κB (p65) binded to the kappa-B consensus sequence and further promoted the transcription of pro-inflammatory genes [38]. It was increasingly recognized that the activation of NF-κB is mainly mediated by IRE1 which could bind to IκB kinase (IKK) and further indirectly activate NF-κB via phosphorylating IκB [39]. In our study, the expressions and activation of three ER stress transducers (PERK, ATF6 and IRE1) were detected in the kidney of septic rats, it was shown that the expression of activated IRE1 increased about 4.73 folds which was significantly higher than those of activated PERK and ATF6, suggesting that IRE1-mediated signaling pathway was mainly responsible for the development of septic AKI. Interestingly, tunicamycin which was a classic inducer of ER stress displayed similar effects as LPS on the HK-2 cells. It enhanced the phosphorylation of IRE1 and NF-κB (p65), while both resveratrol and IRE specific inhibitor irestatin 9389 could obviously reverse these deleterious effects and inhibit the phosphorylation of IRE1 and NF-κB (p65). Taken together, the inhibition of ER stress (mainly IRE1 pathway) reduced the activation of NF-κB (p65) and attenuated the inflammatory response in the renal tubular epithelial cells during sepsis, it might contribute to the protective effect of resveratrol on septic AKI.

In summary, our data demonstrated that IRE1-NF-κB pathway was obviously activated by ER stress during sepsis and contributed to the septic AKI through enhancing the production of pro-inflammatory cytokines (TNF-α, IL-1β and IL-6) in the renal proximal tubular epithelial cells. Administration of resveratrol as soon as possible after the onset of sepsis could ameliorate septic AKI through inhibiting renal inflammatory response triggered by ER stress-activated IRE1-NF-κB pathway. Therefore, resveratrol might be a readily translatable option to improve the prognosis of sepsis.

## MATERIALS AND METHODS

### Animal

Male SD rats weighed 180–200 g were purchased from the Center of Experimental Animals in Central South University of China. The rats were kept on a 12-h light/dark cycle and housed individually with free access to food and water throughout the experiment. Animal use procedures were approved by the animal welfare ethics committee of Central South University. The contract numbers of approval documents was 20151801.

### Establishment of a rat model of polymicrobial sepsis and treatment

A rat model of sepsis was established by CLP. Before operation, the rats were fasted for 10–12 hours. According to the process we previously reported [40], rats were weighed and anesthetized by 10% chloral hydrate through intraperitoneal injection, then they underwent laparotomy and ligation of the cecum just below the ileocecal valve with double punctures(18-gauge needle) at the antimesenterial wall for three times. Thereafter, the abdominal wall was closed in two layers. 5 mL/kg of 0.9% NaCl was injected subcutaneously after operation, food and water were provided *ad libitum*.

The rats were randomly divided into four groups as follows: (1) sham-operated control group (Sham); (2) sham-operated group with resveratrol treatment (Sham+Res); (3) CLP group; (4) CLP group with resveratrol treatment (CLP+Res). Rats in the sham group and sham+Res group underwent laparotomy without CLP. Resveratrol was dissolved in DMSO to a final concentration of 20 mg/ml (resveratrol:DMSO:H_2_O=3:5:150) and administrated immediately after CLP operation through intraperitoneal injection at 30 mg/kg, while rats in the sham group and sham+Res group were given equal volume of 0.9% NaCl contained the same concentration of DMSO as resveratrol. Except the rats for survival test, rats were sacrificed at 6 or 12 hours after the operation.

### Reagents

The antibodies to Bip/GRP78, PERK, phospho-PERK (Thr980), phospho-NF-κB(Ser536), GAPDH were purchased from cell signaling technology. The antibodies to IRE1, phospho-IRE1(Ser724) and NF-κB (p65) were purchased from Abcam. Rabbit polyclonal ATF6α (H-280) antibody was purchased from Santa Cruz. Tunicamycin, resveratrol, lipopolysaccharide were purchased from Sigma-Adrich. TNF-α, IL-1β and IL-6 ELISA kits and IgG-horseradish peroxidase conjugated second antibody were purchased from Boster. Irestatin 9389 was purchased from Axon Medchem. Other reagents were analytically pure.

### Histological examination

Kidney tissues were rinsed and fixed in 4% paraformaldehyde (PFA) at 4°C overnight. The PFA-treated kidney tissues were then processed with sequential clearing and dehydrating steps, followed by embedding in the paraffin blocks. Samples were sectioned into 5 μm slices and subjected to standard Hematoxylin and Eosin (H&E) staining for the evaluation of kidney tissue injury. Images were taken under an optical microscope (Olympus).

### Measurement of serum BUN and Cr levels

Blood was obtained by direct cardiac puncture under deep anesthesia at 12 h after CLP operation and clot at room temperature for 4 h. To get serum samples, the blood was centrifuged at 1,500 g for 10 min. The serum BUN and Cr levels were further detected by colorimetric method.

### Immunohistochemistry

Kidney sections were dewaxed in xylene and rehydrated through graded ethanols. Then antigen retrieval was performed by immersing the sections in the citrate buffer (PH 6.0) at 80°C for 2 hours. After natural cooling, the sections were washed 2 times by PBS for 5 minutes each time and were immersed in 0.3% Triton X-100 for 15 minutes. After blocking by 1% bovine serum albumin, the sections were incubated with NF-κB (p65) antibody in 1% bovine serum albumi(1:25) at 4°C overnight and then washed 3 times by PBS. IgG-horseradish peroxidase conjugated second antibody was then used to incubate the sections for 1 hour at room temperature. The location of NF-κB (p65) in the kidney was visualized by 3, 3′-diaminobenzidine (DAB). Sections were washed 3 times by PBS followed by counterstaining with hematoxylin for 1–3 minutes, dehydrating in 100% ethanol, clearing in xylene, mounting with neutral balsam and finally photographing under an optical microscope (Olympus). 5 random fields per section were analyzed by Image pro-Plus 7.0 sofeware to calculate the percentage of nuclear NF-κB(p65)-positive cells in the kidney.

### Western blotting

Kidney tissues or HK-2 cells were homogenized or scraped with the lysate buffer as previously described[41]. Homogenized samples were centrifuged at 4°C, 14,000 rpm for 30 min; cells were scraped with lysate and centrifuged at 4°C, 14,000 rpm for 10 min. The concentrations of protein from the supernatant were determined by BCA protein assay reagent. 20~100 μg protein was loaded to 10% SDS polyacrylamide gel electrophoresis and then transferred to polyvinylidene difluoride membranes (Millipore, USA). After blocking with 5% bovine serum albumin, immunoblotting was carried out with different primary antibodies at 4°C overnight or at 25°C for 2 hours (anti-GAPDH antibody was used as internal controls). After incubation with IgG-horseradish peroxidase conjugated second antibody at room temperature for 1 h, membranes were washed three times successively. Then the specific proteins were detected by enhanced or super chemiluminescence (Invitrogen, USA) according to the manufacture's instruction. The relative band intensity was quantified by Quantity One software.

### Quantitative real-time polymerase chain reaction

Total RNA of the kidney tissues and HK-2 cells were extracted by trizol and reverse transcribed to cDNA with Primescript^TM^ RT reagent kit with gDNA eraser according to the manufacturer's instructions (Takara shuzo Co., Kyoto, Japan). The concentration and purity of total RNA were determined by measuring the OD260 and OD260/OD280 ratio respectively. The mRNA expressions were measured by SYBRR Premix Ex Taq^TM^ (Takara shuzo Co., Kyoto, Japan) through an ABI 7500 real-time PCR system(Life Technology Corporation, Carsbad, CA). Each cDNA sample was done in triplicate. The relative quantitation of mRNA was analyzed using the equation as follows: Ratio = 2^−△△Ct^ and normalized by GAPDH. The following primers for rats were used: TNF-α-forward: 5′-TACTGAACTTCGGGGTGATCG-3′ and reverse: 5′-TCCGCTTGGTGGTTTGCTAC-3′; IL-1β-forward: 5′-GACAAGCAACGACAAAATCCC-3′ and reverse: 5′-GAAGACAAACCGCTTTTCCATC-3′; IL-6-forward 5′-CGAAAGTCAACTCCATCTGCC-3′ and reverse: 5′-TGTTGTGGGTGGTATCCTCTG-3′; Hemo oxygenase-1(HO-1)-forward: 5′-AGCGAA ACAAGCAGAACCCA-3′ and reverse: 5′-ACCTCGT GGAGACGCTTTAC-3′; Kidney injury molecular-1(KIM-1)-forward: 5′-GGGTCTCCTTCACAGCACATT-3′ and reverse: 5′-AAGCACTGGGTACAGATCCAAA-3′; Neutrophil gelatinase-associated lipocalin (NGAL)-forward: 5′-GGAAGAACCAAGGGGCTGTC-3′ and reverse: 5′-CGCTCACCGTCTGTTCAGTT-3′; GAPDH-forward: 5′-AAGCCCATCACCATCTTCCA-3′ and reverse: 5′-CCTGCTTCACCACCTTCTTG-3′. Primers for human were as follows: TNF-α-forward: 5′-CC CATCTATCTGGGAGGGGT-3′ and reverse: 5′-GC GTTTGGGAAGGTTGGATG-3′; IL-1β-forward: 5′-AGC TCGCCAGTGAAATGATG-3′ and reverse: 5′-GTAG TGGTGGTCGGAGATTCG-3′; IL-6-forward 5′-TCA ATATTAGAGTCTCAACCCCCAA-3′ and reverse: 5′-CAGGGAGAAGGCAACTGGAC-3′; GAPDH-forward: 5′- AATGGGCAGCCGTTAGGAAA-3′ and reverse: 5′-GCGCCCAATACGACCAAATC-3′.

### Enzyme-linked immunoabsorbent assays (ELISA)

ELISA assays were performed to detect the TNF-α, IL-1β and IL-6 contents in serum or cell supernatant according to the manufacture′s protocols.

### Cell culture

An immortalized proximal tubule epithelial cell line from normal adult human kidney (HK-2) was purchased from the Cell Bank of the Chinese Academy of Sciences (Shanghai, China). All cells were cultured in F-12/DMEM medium (Hyclone, USA) supplemented with 10% fetal bovine serum (Hyclone, USA), 100 U/ml penicillin, 100 μg/ml streptomycin, 5 ng/mL recombinant epidermal growth factor (Sigma-Adrich) at 37°C under a humidified atmosphere of 95% air with 5% CO_2_ to allow the cells to grow and form a monolayer in the flask. After confluence, cells were trypsinized using a 0.25% trypsin solution in Hanks buffer for 2 min and resuspended in complete culture medium. 1 μg/ml lipopolysaccharide was used to set up an inflammatory condition *in vitro*.

### MTT assay

The cell viability was detected by MTT(3-[4, 5-2-yl]-2, 5-diphenyltetrazolium bromide) method as previously decribed [41]. Data were shown as the percentage of viable cells compared with the control group which was considered as 100%.

### Statistical analysis

All data were analyzed by SPSS 18.0 software. Measurement data were shown as mean ± S.E.M of three different experiments and analyzed by unpaired two-tailed Student′s *t* tests. Kaplan-Meier analysis was performed to compare the differences in survival rate between different groups. *P* < 0.05 was considered statistically significant.

## Abbreviations

AKI: acute kidney injury
MODS: multiple organ dysfunctions
OD: organ dysfunction
LPS: lipopolysaccharide
CLP: cecal ligation puncture
TNF-α: tumor necrosis factor-α
IL-1β: interleukin-1β
IL-6: interleukin-6
NF-κB: nuclear transcription factor-κB
IRE1: inositol-requiring enzyme 1
ER: endoplasmic reticulum
HO-1: Heme Oxygenase-1
KIM-1: kidney injury molecular-1
NGAL: neutrophil gelatinase-associated lipocalin
IKK: IκB kinase

## References

[1] De Suarez La, Rica A, Gilsanz F, Maseda E Epidemiologic trends of sepsis in western countries. Ann Transl Med.

[2] Liao X, Du B, Lu M, Wu M, Kang Y (2016). Current epidemiology of sepsis in mainland China. Ann Transl Med.

[3] Martensson J, Bellomo R (2015). Sepsis-Induced Acute Kidney Injury. Crit Care Clin.

[4] Gomez H, Ince C, De Backer D, Pickkers P, Payen D, Hotchkiss J, Kellum JA (2014). A unified theory of sepsis-induced acute kidney injury: inflammation, microcirculatory dysfunction, bioenergetics, and the tubular cell adaptation to injury. Shock.

[5] Smith JA, Mayeux PR, Schnellmann RG (2016). Delayed Mitogen-Activated Protein Kinase/Extracellular Signal-Regulated Kinase Inhibition by Trametinib Attenuates Systemic Inflammatory Responses and Multiple Organ Injury in Murine Sepsis. Crit Care Med.

[6] Alter P, Rupp H, Maisch B (2006). Activated nuclear transcription factor kappaB in patients with myocarditis and dilated cardiomyopathy—relation to inflammation and cardiac function. Biochem Biophys Res Commun.

[7] Zhang D, Cai Y, Chen M, Gao L, Shen Y, Huang Z (2015). OGT-mediated O-GlcNAcylation promotes NF-kappaB activation and inflammation in acute pancreatitis. Inflamm Res.

[8] Zhang K, Jiao XF, Li JX, Wang XW (2015). Rhein inhibits lipopolysaccharide-induced intestinal injury during sepsis by blocking the toll-like receptor 4 nuclear factor-kappaB pathway. Mol Med Rep.

[9] Yu C, Qi D, Sun JF, Li P, Fan HY (2015). Rhein prevents endotoxin-induced acute kidney injury by inhibiting NF-kappaB activities. Sci Rep.

[10] Pahl HL, Baeuerle PA (1995). A novel signal transduction pathway from the endoplasmic reticulum to the nucleus is mediated by transcription factor NF-kappa B. EMBO J.

[11] Ma T, Han L, Gao Y, Li L, Shang X, Hu W, Xue C (2008). The endoplasmic reticulum stress-mediated apoptosis signal pathway is involved in sepsis-induced abnormal lymphocyte apoptosis. Eur Surg Res.

[12] Endo M, Oyadomari S, Suga M, Mori M, Gotoh T (2005). The ER stress pathway involving CHOP is activated in the lungs of LPS-treated mice. J Biochem.

[13] Esposito V, Grosjean F, Tan J, Huang L, Zhu L, Chen J, Xiong H, Striker GE, Zheng F (2013). CHOP deficiency results in elevated lipopolysaccharide-induced inflammation and kidney injury. Am J Physiol Renal Physiol.

[14] Chen L, Yang S, Zumbrun EE, Guan H, Nagarkatti PS, Nagarkatti M (2015). Resveratrol attenuates lipopolysaccharide-induced acute kidney injury by suppressing inflammation driven by macrophages. Mol Nutr Food Res.

[15] Holthoff JH, Wang Z, Seely KA, Gokden N, Mayeux PR (2012). Resveratrol improves renal microcirculation, protects the tubular epithelium, and prolongs survival in a mouse model of sepsis-induced acute kidney injury. Kidney Int.

[16] Lin Y, Zhu J, Zhang X, Wang J, Xiao W, Li B, Jin L, Lian J, Zhou L, Liu J (2016). Inhibition of Cardiomyocytes Hypertrophy by Resveratrol Is Associated with Amelioration of Endoplasmic Reticulum Stress. Cell Physiol Biochem.

[17] Pan QR, Ren YL, Liu WX, Hu YJ, Zheng JS, Xu Y, Wang G (2015). Resveratrol prevents hepatic steatosis and endoplasmic reticulum stress and regulates the expression of genes involved in lipid metabolism, insulin resistance, and inflammation in rats. Nutr Res.

[18] Lou Y, Wang Z, Xu Y, Zhou P, Cao J, Li Y, Chen Y, Sun J, Fu L (2015). Resveratrol prevents doxorubicin-induced cardiotoxicity in H9c2 cells through the inhibition of endoplasmic reticulum stress and the activation of the Sirt1 pathway. Int J Mol Med.

[19] Singer M, Deutschman CS, Seymour CW, Shankar-Hari M, Annane D, Bauer M, Bellomo R, Bernard GR, Chiche JD, Coopersmith CM, Hotchkiss RS, Levy MM, Marshall JC (2016). The Third International Consensus Definitions for Sepsis and Septic Shock (Sepsis-3). JAMA.

[20] Cunningham PN, Dyanov HM, Park P, Wang J, Newell KA, Quigg RJ (2002). Acute renal failure in endotoxemia is caused by TNF acting directly on TNF receptor-1 in kidney. J Immunol.

[21] Kolgazi M, Sener G, Cetinel S, Gedik N, Alican I (2006). Resveratrol reduces renal and lung injury caused by sepsis in rats. J Surg Res.

[22] Sebai H, Ben-Attia M, Sani M, Aouani E, Ghanem-Boughanmi N (2008). Protective effect of resveratrol on acute endotoxemia-induced nephrotoxicity in rat through nitric oxide independent mechanism. Free Radic Res.

[23] Zager RA, Johnson AC, Becker K (2012). Plasma and urinary heme oxygenase-1 in AKI. J Am Soc Nephrol.

[24] Chandrashekhara HB, Shariff MG, Ns R (2016). Diagnostic value of plasma NGAL to predict acute kidney injury in patients with suspected sepsis admitted to emergency and MICU. J Assoc Physicians India.

[25] Bonventre JV (2008). Kidney Injury Molecule-1 (KIM-1): a specific and sensitive biomarker of kidney injury. Scand J Clin Lab Invest Suppl.

[26] Wang P, Huang J, Li Y, Chang R, Wu H, Lin J, Huang Z (2015). Exogenous Carbon Monoxide Decreases Sepsis-Induced Acute Kidney Injury and Inhibits NLRP3 Inflammasome Activation in Rats. Int J Mol Sci.

[27] Luo CJ, Luo F, Zhang L, Xu Y, Cai GY, Fu B, Feng Z, Sun XF, Chen XM (2016). Knockout of interleukin-17A protects against sepsis-associated acute kidney injury. Ann Intensive Care.

[28] He L, Peng X, Zhu J, Chen X, Liu H, Tang C, Dong Z, Liu F, Peng Y (2014). Mangiferin attenuate sepsis-induced acute kidney injury via antioxidant and anti-inflammatory effects. Am J Nephrol.

[29] Wilkinson R, Wang X, Roper KE, Healy H (2011). Activated human renal tubular cells inhibit autologous immune responses. Nephrol Dial Transplant.

[30] Marko L, Vigolo E, Hinze C, Park JK, Roel G, Balogh A, Choi M, Wubken A, Cording J, Blasig IE, Luft FC, Scheidereit C, Schmidt-Ott KM (2016). Tubular Epithelial NF-kappaB Activity Regulates Ischemic AKI. J Am Soc Nephrol.

[31] Meng XM, Nikolic-Paterson DJ, Lan HY (2014). Inflammatory processes in renal fibrosis. Nat Rev Nephrol.

[32] Zhang P, Li Y, Zhang LD, Wang LH, Wang X, He C, Lin ZF (2014). Proteome changes in mesenteric lymph induced by sepsis. Mol Med Rep.

[33] Song J, Hu D, He C, Wang T, Liu X, Ma L, Lin Z, Chen Z (2013). Novel biomarkers for early prediction of sepsis-induced disseminated intravascular coagulation in a mouse cecal ligation and puncture model. J Inflamm (Lond).

[34] Zhang C, Lin G, Wan W, Li X, Zeng B, Yang B, Huang C (2012). Resveratrol, a polyphenol phytoalexin, protects cardiomyocytes against anoxia/reoxygenation injury via the TLR4/NF-kappaB signaling pathway. Int J Mol Med.

[35] Feng Y, Cui Y, Gao JL, Li MH, Li R, Jiang XH, Tian YX, Wang KJ, Cui CM, Cui JZ (2016). Resveratrol attenuates neuronal autophagy and inflammatory injury by inhibiting the TLR4/NF-kappaB signaling pathway in experimental traumatic brain injury. Int J Mol Med.

[36] Li J, Xie C, Zhuang J, Li H, Yao Y, Shao C, Wang H (2015). Resveratrol attenuates inflammation in the rat heart subjected to ischemia-reperfusion: Role of the TLR4/NF-kappaB signaling pathway. Mol Med Rep.

[37] Adamzik M, Schafer S, Frey UH, Becker A, Kreuzer M, Winning S, Frede S, Steinmann J, Fandrey J, Zacharowski K, Siffert W, Peters J, Hartmann M (2013). The NFKB1 promoter polymorphism (−94ins/delATTG) alters nuclear translocation of NF-kappaB1 in monocytes after lipopolysaccharide stimulation and is associated with increased mortality in sepsis. Anesthesiology.

[38] Tanaka M, Fuentes ME, Yamaguchi K, Durnin MH, Dalrymple SA, Hardy KL, Goeddel DV (1999). Embryonic lethality, liver degeneration, and impaired NF-kappa B activation in IKK-beta-deficient mice. Immunity.

[39] Zhang K, Kaufman RJ (2008). From endoplasmic-reticulum stress to the inflammatory response. Nature.

[40] Gao M, Zhang L, Liu Y, Yang M, Wang N, Wang K, Ou D, Liu M, Chen G, Liu K, Xiao X (2012). Use of blood urea nitrogen, creatinine, interleukin-6, granulocyte-macrophage colony stimulating factor in combination to predict the severity and outcome of abdominal sepsis in rats. Inflamm Res.

[41] Wang N, Zhang D, Mao X, Zou F, Jin H, Ouyang J (2009). Astragalus polysaccharides decreased the expression of PTP1B through relieving ER stress induced activation of ATF6 in a rat model of type 2 diabetes. Mol Cell Endocrinol.

